# Instruments for assessing patient-reported experience measures among patients with diabetes mellitus: a scoping review

**DOI:** 10.1186/s41687-025-00848-7

**Published:** 2025-02-08

**Authors:** Soe Sandi Tint, Myo Zin Oo, Nida Buawangpong, Wichuda Jiraporncharoen, Nutchar Wiwatkunupakarn, Kittipan Rerkasem, Kanokwan Kulprachakarn, Hataichanok Chuljerm, Timothy E. O’Brien, Rohini Mathur, Chaisiri Angkurawaranon

**Affiliations:** 1 Global Health and Chronic Conditions Research Center, Chiang Mai University, Chiang Mai, 50200 Thailand; 2 Department of Family Medicine, Faculty of Medicine, Chiang Mai University, 110 Inthawarorot Rd., Sriphum, Muang, Chiang Mai, 50200 Thailand; 3 School of Health Sciences Research, Research Institute for Health Sciences, Chiang Mai University, Chiang Mai, Thailand; 4 Environmental-Occupational Health Sciences and Non-Communicable Diseases Research Center, Research Institute for Health Sciences, Chiang Mai University, Chiang Mai, Thailand; 5 Clinical Surgical Research Center, Department of Surgery, Faculty of Medicine, Chiang Mai University, Chiang Mai, Thailand; 6 Department of Mathematics and Statistics, Loyola University Chicago, Chicago, IL 60660 USA; 7 Wolfson Institute for Population Health, Queen Mary University of London, London, E1 2AT UK

**Keywords:** Diabetes mellitus, Patient-reported experience measures, Patient-centered care, Patient outcome assessment

## Abstract

**Purpose:**

Diabetes Mellitus (DM) management is increasingly focusing on patient-centered care, making patient-reported experience measures (PREMs) critical for understanding the subjective aspects of diabetes treatment and self-management. These measures differ based on cultural contexts and individual perspectives, leading different countries to the development of country-specific tools to assess care quality from the patient’s viewpoint. This review aimed to identify available instruments for assessing patient-reported experiences in individuals with diabetes and examine the different domains, items, and the validity and reliability of these instruments.

**Methods:**

Following PRISMA-ScR guidelines, databases including PubMed, Embase, CINAHL, Cochrane, and Scopus were searched for English-language articles without year limitations. This scoping review focused on PREMs that evaluate the quality of diabetes care among adolescent and adult patients with type 1 and type 2 DM. Studies that used patient expectation questionnaires, involved individuals not receiving care, or focused on patient-reported outcomes rather than experiences were excluded.

**Results:**

Eight articles from six countries representing different healthcare settings were included, mostly from developed countries. A variety of methodologies were used to develop these PREM instruments, with unique domains and items. Content analysis revealed five commonly measured domains: (1) care planning, (2) patient education, (3) professionalism, (4) quality of care, and (5) hospital care and transition, reflecting diverse patient experiences across healthcare services.

**Conclusions:**

This scoping review identifies a limited number of tools for evaluating PREMs in diabetes care, highlighting variability in their development and domain coverage. Five core domains are proposed across different settings, with an emphasis on culturally adapted measures to enhance the accuracy of patient experience capture in diverse populations.

**Supplementary Information:**

The online version contains supplementary material available at 10.1186/s41687-025-00848-7.

## Introduction

Diabetes Mellitus (DM) is a chronic disease, and its prevalence has been rising globally, with significant health and economic implications. According to the World Health Organization (WHO), the number of adults living with diabetes exceeded 400 million worldwide in 2019 [1]. High-quality care is essential to optimize health outcomes, enhance quality of life, and reduce the burden of diabetes-related complications [2]. The American Diabetes Association defines quality care as encompassing effectiveness, safety, and patient-centeredness, which includes timely diagnosis, appropriate management of blood glucose levels, complication prevention and management, patient education, and shared decision-making between healthcare providers and patients [3].

A fundamental component of quality diabetes care is patient-centered care, which aligns healthcare services with patients’ needs, preferences, and values [4]. Central to patient-centered care is the concept of patient-reported experience, which encompasses “the range of interactions that patients have with the healthcare system, including their care from doctors, nurses, and staff in hospitals, physician practices, and other healthcare facilities” [5]. The patient experience reflects aspects of care that patients prioritize, such as timely appointments, access to information, and effective communication with healthcare providers [6]. Therefore, understanding and improving patient experience is essential for ensuring that diabetes care is truly patient-centered.

Patient-Reported Experience Measures (PREMs) are tools used to assess patients’ subjective experiences regarding their care and interactions with healthcare services [7]. Unlike Patient-Reported Outcome Measures (PROMs), which capture health outcomes such as symptom burden and quality of life from the patient’s perspective [7], PREMs specifically focus on patients’ experiences with healthcare delivery, including communication, access, and satisfaction with care processes. This differentiation is essential, as PROMs reflect what patients experience regarding health status, whereas PREMs capture how patients experience their care interactions. Both of these measures are instrumental in assessing the extent to which care is patient-centered. However, general PREMs often fail to capture the unique aspects of care required by patients with chronic conditions like diabetes. Diabetes management is complex, involving specific concerns such as blood glucose monitoring, medication management, lifestyle modifications, and the prevention of complications like hypoglycemia and diabetic ketoacidosis. These factors necessitate the use of diabetes-specific PREMs that address the particular needs and challenges of diabetes care [8].

Existing general tools, such as the Instrument for Evaluation of the Experience of Chronic Patients **(IEXPAC)** [9], the Patient Perception for Chronic Illness Care **(PPCI)** [8] and brief questionnaire **(PEQ)** developed in primary health care for measuring patients’ experience of interaction, emotion and consultation outcome [10], are valuable in assessing the overall experience of patients with chronic conditions. However, these instruments are not tailored to capture the unique experiences of diabetes patients. For instance, IEXPAC focuses broadly on chronic diseases but does not provide detailed insights into the specific aspects of diabetes management, such as the patient’s experience with blood glucose control or diabetes-related complications [9]. Similarly, the PPCI is designed to assess perceptions of chronic illness care in general, but it does not delve deeply into the nuances of diabetes care, such as the impact of personalized care plans, diabetes education, and the integration of self-management into daily life Also, the PEQ tool was developed mainly for consultation-specific assessment rather than chronic disease [9, 11] management. Therefore, while these tools offer useful general information, they lack the specificity needed to comprehensively assess diabetes-related care and experiences.

This gap underscores the importance of developing and utilizing diabetes-specific PREMs, particularly in settings where diabetes care plays a pivotal role, including inpatient, outpatient, and primary care contexts. Diabetes patients face unique challenges in managing their condition, even when their hospitalization or treatment may be for other health concerns [3, 11, 12]. Tools specifically designed for diabetes care capture the critical elements of the patient experience, such as the management of diabetes-related risks, communication with healthcare providers about self-management, and involvement in decision-making regarding their care. By focusing on diabetes-specific instruments, this review aims to highlight the tools that can provide a deeper understanding of the patient experience in diabetes care, helping to identify areas for improvement and support the development of targeted interventions. In the context of diabetes management, PREMs can provide valuable insights into how patients perceive the quality of care they receive and highlight areas where healthcare delivery aligns with or falls short of patient expectations [13]. Furthermore, previous studies have shown that patients who are engaged in their care and decision-making are more likely to adhere to medical recommendations and self-care activities [13]. Thus, PREMs can drive continuous improvement in diabetes care by identifying strengths and areas for enhancement.

However, developing instruments to measure PREMs is challenging due to diverse cultural contexts and individual perspectives. Different countries and healthcare systems may require tailored tools to assess the quality of care accurately from the patient’s viewpoint [14]. These variations can lead to differing domains and items within PREMs, potentially impacting their generalizability across settings. Given the cultural diversity and the distinct patient experiences that exist, PREMs developed in one context may not be directly applicable in another, which could limit their utility in new environments [15]. Therefore, it is essential to address these concerns regarding the generalizability of PREMs to ensure they are relevant and effective across different populations and healthcare settings. Understanding the limitations of existing instruments can inform potential adaptations for diverse patient groups. However, there remains a lack of comprehensive understanding of the PREMs currently used in diabetes care across various contexts.

A scoping review can help address these issues by mapping existing PREMs used in diabetes care, identifying the domains and items reported in the literature, and highlighting existing gaps [16]. This review specifically aims to identify instruments designed to assess patient-reported experiences among diabetes patients, investigate and summarize common domains and items used, and report the validity and reliability of the tools currently used in the literature. By understanding the limitations and generalizability of these instruments, we can better inform potential adaptations for diverse patient groups. Ultimately, this comprehensive understanding of the instruments used to measure PREMs will advance diabetes care by effectively capturing patient-centered measures and laying the groundwork for future development of culturally relevant PREMs tailored to specific populations.

## Methods

This scoping review was conducted to identify instruments designed to assess patient-reported experiences among diabetes patients, following the PRISMA-ScR guidelines [17]. The methodology involved a systematic literature search across five databases, the application of pre-defined inclusion and exclusion criteria, and a qualitative synthesis of the findings. This approach allowed for a comprehensive assessment of existing instruments, providing insights into their development and applicability in various healthcare contexts.

### Study selection

#### Inclusion criteria

This scoping review followed the Extension for Scoping Reviews (PRISMA-ScR) checklists [17] Supplementary material 1. The review focuses exclusively on disease-specific PREMs to ensure that the instruments accurately capture the unique experiences and challenges faced by diabetes patients. Disease-specific tools are designed to address the particular aspects of care relevant to diabetes, allowing for more meaningful insights into patient experiences and ultimately supporting improved patient outcomes in this population. It includes studies focusing on PREMs designed for adolescents and adults with Type 1 Diabetes (T1D) or Type 2 Diabetes (T2D). Instruments that assess patient-reported experiences related to diabetes care services, including those used in hospitals, outpatient care clinics, and diabetes care clinics, are included. Instruments that cover a broader age range included if they provide relevant data for adolescents and adults, with specific data extracted and analyzed for these age groups.

#### Exclusion criteria

Articles focusing exclusively on pediatric diabetes patients (under 12 years old) were excluded because they primarily rely on proxy measures from parents or caregivers. We aimed to gather direct insights from adolescents and adults, who can articulate their experiences independently. Studies assessing patient expectations were excluded to focus specifically on experiences with diabetes care services. While expectations are relevant, they can differ significantly from actual experiences. By concentrating on experiences, we aimed to capture direct insights into patient interactions with their care, providing a clearer understanding of care effectiveness and areas for improvement [18]. Articles focusing on patient-reported outcomes rather than experiences with diabetes care services are also excluded because of their difference in outcome measures as highlighted in the introduction [7]. Additionally, articles that focused solely on the development without testing the instrument on patients with diabetes or those not specifically addressing T1D or T2D were excluded to ensure relevance to the experiences of patients with these conditions.

### Information sources and search strategy

The systematic search was independently conducted by two researchers (SSDT, MZO), and articles were searched from five commonly used databases: PubMed, Scopus, Cochrane, CINAHL, and EMBASE. All free full-text journal articles published in English were included in this review, regardless of the year the study was conducted. A combination of Medical Subject Headings (MeSH) and free-texts were used. Two groups of terms were generated to describe the studied population and PREM. For example, the search strategy for PubMed: [Type 1 diabetes mellitus [Mesh] OR Type 2 diabetes mellitus [Mesh]] “AND” [“patient-reported experience” OR “patient-reported experience measures” OR “PREM”]. The full search terms are provided in Supplementary material 2.

### Study screening

All the references from the five databases (*n* = 355) were imported into Rayyan, a web-based tool for systematic reviews [19] where duplicates were removed before the title and abstract screening for relevance to this scoping review. Titles and abstracts were independently reviewed by two reviewers (SSDT, MZO) to identify eligible criteria. Reviewers met throughout the screening process to discuss any uncertainties related to study selection. After title and abstract screening, each author independently reviewed and labeled all the articles and met to resolve any conflicts, ensuring consistency between the reviewers and the research question and purpose.

### Data charting/collection/extraction

Data extraction from each study was performed by two authors (SDT, MZO). The sheet included the title of the review article, the year of publication, the year the study was conducted, the type of study, the number of domains and items, the type of diabetes, the target population, mode of administration, recall period, number of participants, response options, range of scores, and original language. The domains and items used to assess the different domains from each study and information about the validation procedure used in each instrument were also included.

### Summarizing common domains used in PREM instruments

It is expected that different instruments for PREM may assess different domains. To summarize the different domains used in PREM instruments, researchers recorded all domains and items (questions) for each PREM instrument. The authors (SDT, WJ, NB, CA) then used content analysis on these original domains and items to summarize commonly used domains into a coherent and manageable set of synthesized domains [20]. The content analysis followed the following four steps.

#### Decontextualization

Researchers transcribed extracted domains and items from each instrument to familiarize themselves with the data. This step involved identifying initial themes and selecting specific items that represented diverse patient experiences.

#### Recontextualization

The extracted data was examined for recurring patterns and key terms, which encapsulated core patient experiences. In the coding phase, different parts of the data were labeled with short phrases or “codes” that described their main ideas, which were then grouped into categories to identify patterns and connections.

#### Categorization

The original domains were mapped into the developed themes, ensuring that each theme accurately reflects the content and intent of the domains. This step involved refining the themes to verify their relevance and coherence in representing patient perceptions.

#### Compilation

Finally, a summary of commonly used domains in PREMs was identified and given descriptive names that capture their core meanings.

### Psychometric properties of PREM instruments

Psychometric properties refer to the attributes that determine a measurement tool’s reliability, validity, and overall quality. *Reliability*: This refers to the consistency of the measurement tool. A reliable PREM will produce similar results under consistent conditions. Common measures of reliability include internal consistency (often assessed using Cronbach’s alpha) and test-retest reliability, which examines the stability of the measure over time. *Validity*: Validity assesses whether the tool measures what it is intended to measure. There are several types of validity: *Face validity*: Indicates that face validity is often considered a subjective judgment of whether an instrument appears to measure what it is intended to. *Content validity*: Ensures the measure covers all relevant aspects of the construct being assessed. *Construct validity and Criterion validity*: Confirms that the measure relates to other measures in ways that are theoretically expected and demonstrate that the measure correlates with a specific outcome or criterion. *Responsiveness*: This is the ability of a measure to detect change over time, particularly in response to an intervention. A responsive PREM can capture improvements or declines in patient experience due to changes in healthcare delivery. The psychometric properties reported are those suggested in the Consensus-based Standards for the Selection of Health Measurement Instruments (COSMIN) guidelines [21].

## Results

**Fig. 1:**
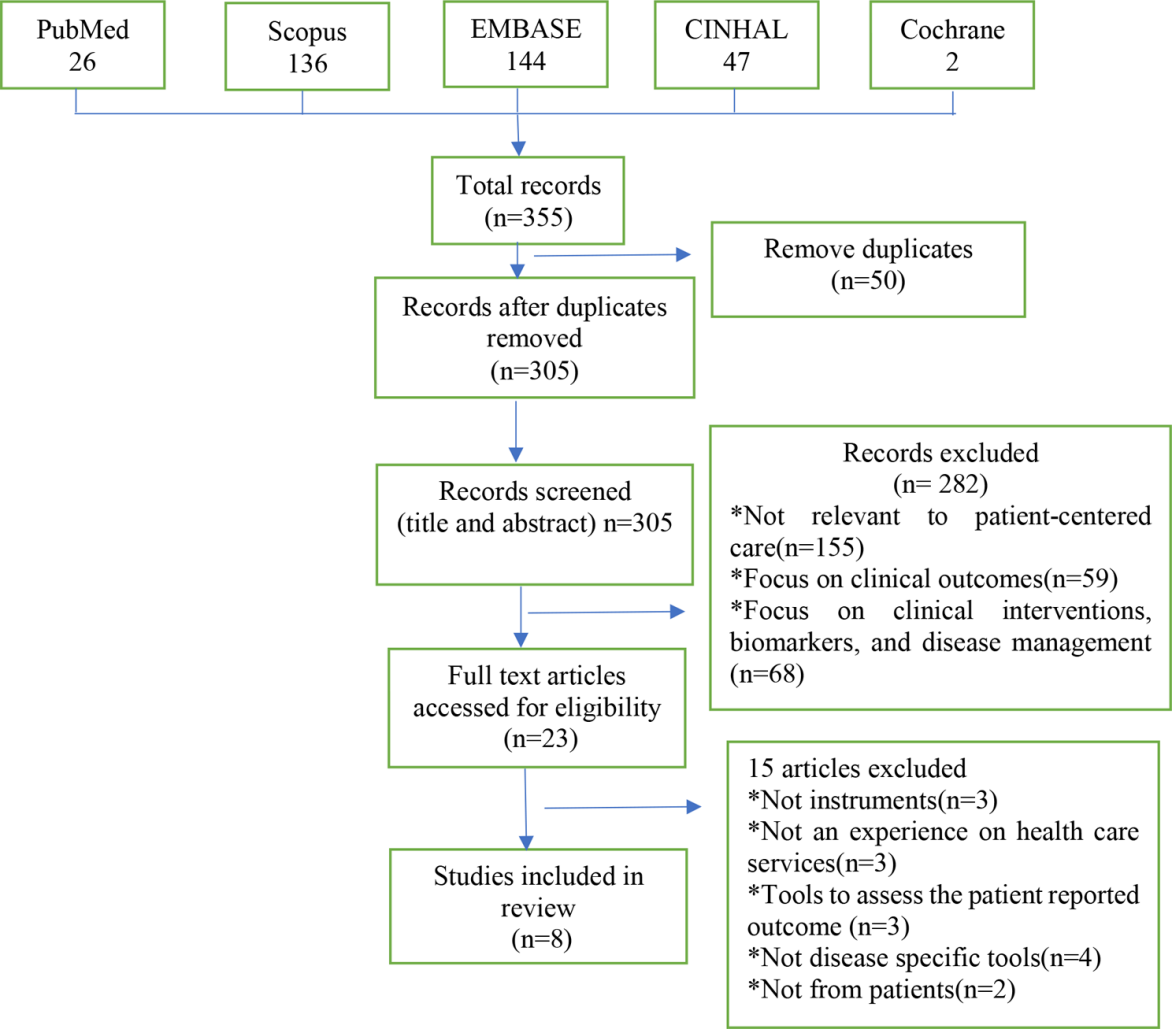
Extension for scoping reviews (PRISMA-ScR) diagram of patient-reported experience measures

Out of a total of 355 records retrieved from five databases, 50 duplicate studies were removed. After the titles and abstracts were reviewed, an additional 282 studies were excluded based on relevance criteria. This left 23 articles for full-text assessment against eligibility criteria, followed by subsequent excluding 15 further studies as they were found to be irrelevant. Figure 1 shows the screening process and reasons for exclusion among the studies identified in the search. Finally, a total of eight articles met the criteria for inclusion in this scoping review. All eight articles described the development of new PREM instruments across five countries. Among these articles, two focused exclusively on Type 1 DM patients, one focused on Type 2 DM patients, and the remaining five focused on both Type 1 and Type 2 DM patients.

### Overview of the instruments

Among the eight articles reviewed, most studies were conducted in high-income countries, including the UK (*n* = 3) [22–24], Sweden (*n* = 1) [25], France (*n* = 1) [26], Norway (*n* = 1) [27], and Denmark (*n* = 1) [28]. One study was conducted in a developing country, Ecuador (*n* = 1) [29]. The publication year ranged from 2014 to 2023. In Sweden and Ecuador, tools for PREMs were developed simultaneously with PROMs for DM patients. In contrast, the tools developed in other countries were focused solely on PREM (*n* = 6). The number of items used in each instrument varied, ranging from 6 to 31. Most of the instruments were targeted for adult populations (*n* = 5) except for those specifically tailored for type 1 DM patients, which were intended for the adolescent population (*n* = 3). Table 1 shows an overview of the instruments.

**Table 1:** Overview of instruments

Author	Instrument	Country	Year	Type	Target population	Diabetes type
The Health and Social Care Information Centre	National Audit, Patient Experience of Diabetes Service Survey (PEDS) [22]	UK	2014	PREM	All ages	1&2
Girling I, et al.	Patient-reported experience measures for young people with type 1 diabetes [23]	UK	2015	PREM	Young people with type 1 DM	1
Svedbo Engström M, et al.	Disease-specific questionnaire for measuring patient-reported outcomes and experiences in the Swedish National Diabetes Register: (NDR-Swedish) [25]	Sweden	2017	PREM & PROM	Adults	1&2
Iversen HH, et al.	The Adolescent Patient Experiences of Diabetes Care Questionnaire (APEQ-DC) [27]	Norway	2019	PREM	Adolescents age 12–17 years	1
Drøjdahl Ryg N, et al.	Patient-reported experience measures for patient satisfaction with outpatient clinic [28]	Denmark	2021	PREM	Adolescent	1
Martin-Delgado J, et al.	Diabetes-specific patient-reported experience and outcome measure (EDP questionnaire [29]	Ecuador	2022	PREM & PROM	All ages	1&2
Kozlowska O, et al.	Patient-reported experience measure for adult inpatient diabetes care [24]	UK	2023	PREM	All ages	1&2
Hehn C, et al.	Type 1 & Type 2 diabetes-specific patient-reported experience measure e-questionnaire: Diabetes-reported experience measures (DREMS) [26]	France	2023	PREM	All ages	1&2

### Characteristics of selected studies

Table 2 presents the selected studies’ detailed characteristics. The sample size of the studies ranged from 177 to 2513. The majority of studies included in the review focused on outpatient diabetes clinic care services (*n* = 5), with one study conducted in an inpatient DM care setting [24], one using national diabetes registry data [22, 25], and another in a primary care services context [29]. Data aimed at assessing their experiences with diabetes care were collected from patients through various methods, using interviews and questionnaires, both online and paper-based methods. Among the studies, three collected data online [22, 24, 26], and three used paper-based methods to provide in-depth feedback on the PREM instrument [23, 28, 29]. Two studies from Sweden and Norway collected PREM data via postal mail [25, 27] (Table 2).

### Psychometric properties of the instruments

Six of the eight instruments were tested for reliability, validity, and responsiveness, whereas the other two did not undergo any such testing [22, 23]. Internal consistency was usually presented as Cronbach’s α (*n* = 4) with values of over > 0.70. The instrument from Ecuador [29] was the only instrument that tested for all of the properties, including internal consistency, test-retest reliability, face validity, content validity, construct validity, and responsiveness. On the other hand, the PREM instrument measures inpatient diabetes care services and has only been tested for responsiveness [24]. Six of the eight instruments included in this review were tested for various psychometric properties such as reliability, validity while two instruments did not undergo any psychometric testing [23, 24]. To provide clarity on how each property was assessed, the methods used for each type of evaluation are detailed below, highlighting the heterogeneity observed across the studies.

### Face validity

Face validity was primarily tested through cognitive interviews or pilot testing with diabetes patients. For example, the instruments from the UK, Sweden, France, and Denmark [25–27, 29] used semi-structured cognitive interviews where participants were asked to think aloud while completing the questionnaire. Probing questions were employed to explore understanding, especially when hesitation or confusion was observed. Participants also provided feedback on the overall relevance, usability, and format of the questionnaire, which was analyzed to inform revisions. The instrument from Ecuador [29] used pilot testing with patient feedback to refine the items.

### Internal consistency

Four instruments reported internal consistency using Cronbach’s α, with acceptable values above 0.70. This measure ensures that the items within the instrument consistently capture the same construct [26–29].

### Test-retest reliability

Test-retest reliability was evaluated using various correlation coefficients, including Intraclass Correlation Coefficient (ICC) [26, 28], weighted kappa [18, 25], and split-half reliability [29]. These methods demonstrated the stability of responses over time.

### Content validity

Nearly all studies’ content validity was assessed through expert panel reviews, which included interdisciplinary teams and diabetes patients. The panels evaluated whether the items comprehensively covered the intended domain and provided feedback for instrument revision [2, 8, 24–26, 28, 29].

### Construct validity

Construct validity was established through techniques such as exploratory or confirmatory factor analysis or by correlating the instrument’s results with other validated tools measuring related constructs [26–29].

### Responsiveness

Responsiveness was assessed in the Ecuador study by evaluating how patient experience scores varied with the number of years since diagnosis. The researchers used the Kruskal–Wallis’s test to compare scores across groups defined by the duration of diabetes [29]. Table 3 includes psychometric properties of each of the selected instruments.

### Summary of common domains in PREMs

Table 4 summarizes the commonly measured domains in PREMs, showing how the original domains from each study align with the five summary domains identified through the content analysis in this review. The detail descriptions of the original domains within each of the five summary domains are describe in Supplementary material 3.

Through the review of the eight PREMs, 29 original domains comprising 110 different items were analyzed. Using content analysis from the domains and each item, the most commonly reported domains of PREMs can be summarized into five domains: (1) care planning, (2) patient education, (3) professionalism, (4) quality of care, and (5) hospital care and transition. The definitions of the summary domains are based on the published literature and are described as follows:

- *Care Planning* is defined as “The process by which health care professionals and patients discuss, agree and review an action plan to achieve the goals or behavior change of most relevance and concern to the patient” [30].
- *Patient Education* is defined as “The process of influencing patient behavior and producing the changes in knowledge, attitudes, and skills necessary to maintain or improve health” [31].
- *Professionalism* is defined as “Commitment to professional competence, honesty with the patient, patient confidentiality, maintaining appropriate relation with the patient, improving quality of care” [32].
- *Quality of Care* is defined as “The degree to which health services for individuals and populations increase the likelihood of desired health outcomes and are consistent with evidence-based professional knowledge. Quality health services should be effective, safe, timely, equitable, integrated, and efficient” [33].
- *Hospital Care and Transition* is defined as “Care provided to patients during hospitalization for diabetes-related issues” [11] and “The transition of care between hospital and home settings (managing diabetes during hospital stays, ensuring continuity of care, and providing support and information for managing diabetes post-discharge)” [34].

**Table 2:** Characteristics of selected studies

No.	Instruments	Total number of questions	Mode of administration	Total no. of participant	Study setting	Types of responses	Original language
1	PEDS [22]	12	Online	714	GP clinics, hospitals, and other specialist services	Yes/No/Not sure	English
2	Patient-reported experience measures for young people with type 1 diabetes [23]	6	Interview	177	Diabetes Clinic	10 point Likert scale (1 = important, 10 = really important)	English
3	NDR-Swedish [25﻿]	12	Mail	972	National diabetes register	-	Swedish
4	APEQ-DC [27]	16	Postal mail	335	Outpatient	5 point Likert scale (1=, not at all, = to a very large extent)	
5	Patient-reported experience measures for patient satisfaction with outpatient clinic [28]	7	interview	357	Outpatient	5 point Likert scale (1=, strongly agree, 5 = strongly disagree)	Norwegian
6	EDP [29]	8	Paper	489	Primary care setting of urban and rural areas	4-point Likert scale (1 = hardly ever, 4 = everyday)	English
7	PREM for adult inpatient care [24]	31	Paper & online	228	Inpatient	Yes/Yes definitely/No/Do not know	
8	DREMS [26]	18	electronic questionnaire	2513	General population with DM	10 point Likert scale (1 = very poor experience, 10 = very good experience)	French

Please refer to Table 1 for the full instrument name

**Table 3:** Psychometric properties of PREM instruments

No.	Instruments	Reliability		Validity			Responsiveness
		Internal consistency	Test-retest	Face validity	Content validity	Construct validity	
1	PEDS [22]	-	-	-	-	-	-
2	Patient-reported experience measures for young people with type 1 diabetes [23]	-	-	-	-	-	-
3	NDR-Swedish [25]	-	Weighted Kappa = 0.78	√ Cognitive interview	√ Expert review panel	-	
4	APEQ-DC [27]	α = 0.75–0.82	-	-	-	√ EFA	-
5	Patient-reported experience measures for patient satisfaction with outpatient [28]	α = 0.81–0.89	ICC value = 0.79	√ Cognitive interview	√ Expert review panel	√ Correlation	
6	EDP [29]	α = 0.81–0.93	Split-halves value = 0.95	√ Pilot test	√ Expert review panel	√ CFA	√ Kruskal–Walli’s test
7	PREM for adult inpatient care [24]	-		√ Cognitive interview	√ Expert review panel	-	
8	DREAMS [26]	α ≥ 0.90	ICC value = 0.79	√ Cognitive interview	√ Expert review panel	√ EFA &CFA	-

Please refer to Table 1 for the full instrument name

α = Cronbach α coefficient

ICC = Intra-class Correlation Coefficient

EFA = Exploratory Factor Analysis

CFA = Confirmatory Factor Analysis

**Table 4:** Summary of commonly measured domains in PREMs

No	Instrument	Original Domains (No. of Items)	Summary of Domains through content analysis						
			Care planning		Patient Education	Professionalism	Quality of service	Hospital care and transition	
1	National Audit, Patient Experience of Diabetes Service Survey (PEDS) [22]	**Care planning (9)**	√			√			
		**Care provision (3)**					√		
2	Patient-reported experience measures for young people with type 1 diabetes [23]	**Learning new information (4)**			√				
		**Age appropriateness (2)**					√		
3	Disease-specific questionnaire for measuring patient-reported outcomes and experiences in the Swedish National Diabetes Register: (NDR-Swedish) [25]	**Support from diabetes care provider (9)**			√		√		
		**Medical devices and medical treatment (3)**	√						
4	The Adolescent Patient Experiences of Diabetes Care Questionnaire (APEQ-DC) [27]	**Consultation (7)**	√		√		√		
		**Information on food and physical activity/exercise (2)**	√		√				
		**Nurse contact (3)**				√			
		**Doctor contact (3)**				√			
		**Outcome (1)**					√		
5	Patient-reported experience measures for patient satisfaction with outpatient [28]	**Satisfaction with the outpatient clinic (1)**					√		
		**Benefit (3)**					√		
		**Accessibility (3)**					√		
6	Diabetes-specific patient-reported experience and outcome measure (EDP questionnaire) [29]	**Information (3)**	√	√					
		**Care delivery (2)**				√	√		
		**Patient-centered care (3)**	√			√			
7	Patient-reported experience measure for adult inpatient diabetes care [24]	**Admission to hospital (2)**	√					√	
		**Managing your diabetes during your hospital stay (4)**						√	
		**Medication and equipment (6)**	√	√				√	
		**Treatment and care (8)**	√			√		√	
		**Communication (5)**					√	√	
		**Hospital food (5)**						√	
		**Leaving hospital (1)**						√	
8	Type 1 & Type 2 diabetes-specific patient-reported experience measure e-questionnaire: Diabetes-reported experience measures (DREMS) [26]	**Relationship with the physician (4)**				√			
		**Medical care experience (4)**	√			√			
		**Illness appropriation (3)**		√					
		**Medical consultation (3)**					√		
		**Care structure (4)**					√		

## Discussion

The concept of patient-centered care is essential for improving the quality and outcomes of care for patients with diabetes. However, there is only a limited number of tools developed to measure PREM for this group. This scoping review highlights variations in the development of PREMs, including differences in domains and items used, and identifies significant gaps in the literature. Despite the recent emergence of research on PREMs since 2014, much of the literature comes from high-income countries, such as the UK, Sweden, France, Norway, and Denmark, with only one study from a middle-income country, Ecuador. The predominance of existing healthcare instruments raises important questions about their applicability and suitability across different healthcare settings. Healthcare challenges, cultural expectations, and patient-provider dynamics can vary significantly across regions, leading to diverse patient experiences. As a result, instruments developed in one context may not fully capture the breadth of experiences in other settings. Future research should prioritize the development and validation of PREMs that are contextually appropriate and tailored to the specific healthcare systems and patient populations of different regions. Such efforts would ensure that these instruments account for local health system challenges, respect cultural differences, and accurately reflect patient experiences across diverse socioeconomic conditions. This would enhance the relevance of PREMs globally and contribute to improving patient-centered care in a variety of healthcare environments.

Moreover, as most DM care and management occur in outpatient settings [35], most studies have examined PREM in outpatient settings. Only one study was conducted in an inpatient DM care setting [24]. The importance of diabetes-specific PREMs is particularly evident in inpatient care. While many diabetes patients are hospitalized for conditions unrelated to diabetes, such as infections or cardiovascular events, diabetes remains a significant factor that influences both their hospitalization experience and their health outcomes [12]. Studies, such as those from the National Diabetes Inpatient Audit (NaDIA) [36] and the National Diabetes Inpatient Safety Audit (NDISA) [37], highlight the increased risks for diabetes patients in hospital settings, including medication errors, severe hypoglycemia, and diabetic ketoacidosis. These risks underscore the need for a comprehensive approach to inpatient diabetes care, one that is informed by feedback from diabetes patients themselves. Diabetes-specific PREMs are essential in this context to capture patient experiences related to their diabetes care, even when it is not the primary reason for their hospitalization. This gap suggests an important direction for future research—developing robust, context-specific PREMs that capture the unique challenges and experiences of inpatient diabetes care. Doing so would help improve the quality of care provided to hospitalized diabetes patients and ensure that their specific needs are being met effectively. Most of the instruments were designed for the adult population (*n* = 5) [22, 24–26, 29] with only three explicitly tailored for adolescents with type 1 DM [23, 27, 28]. This discrepancy points to a potential underrepresentation of the adolescent DM population in the development of these tools. Given that the management and experiences of DM can differ significantly between adults and younger populations, developing age-appropriate instruments is crucial for capturing accurate and meaningful data.

The review identified 29 distinct domains, with the number of items varying significantly, ranging from 6 to 31. This variability in the number of items and domains suggests that the purpose of the PREMs plays a key role in shaping their design. Some instruments are tailored to capture culturally relevant aspects of care, ensuring that the PREMs are responsive to the unique needs of specific patient populations and healthcare environments, as seen in tools from Sweden, Ecuador, and France [25, 26, 29]. In contrast, one instrument from the UK was developed specifically for quality assessment in hospitalized patients, prioritizing aspects of care that are most relevant in that setting [24]. Additionally, some instruments, such as those from Norway and Denmark [27, 28] were designed to assess patient satisfaction in outpatient settings, focusing on domains pertinent to outpatient care or specific treatment experiences. This variation in instrument design could pose challenges in comparing data across different settings and populations, as it reflects differing healthcare contexts, priorities, and patient needs [38]. However, despite these differences, the review also found that many PREMs consistently address common domains such as care planning, care provision, and communication. At the same time, unique focus areas emerged, such as patient experiences with medication, equipment, and specific aspects of doctor-patient communication.

Understanding both the common and unique domains across these instruments can help facilitate comparisons across studies and provide a foundation for developing new PREMs that balance both universal and context-specific aspects of diabetes care. By acknowledging the variability in the purpose and scope of PREMs, future researchers can select the most appropriate tools or develop new ones that align with the specific goals of their studies, whether for quality improvement, patient satisfaction, or cultural adaptation [39].

In reviewing the eight instruments, it was observed that a common methodological approach was employed across most of the studies. Specifically, developing these questionnaires consistently involved direct input from patients and local experts. This method was highlighted in the Swedish study, which focused on tailoring the questionnaire to the Swedish cultural and healthcare environment through qualitative interviews with patients and comments from local healthcare professionals [25]. This method should help ensure that the questionnaires reflect the lived experiences and specific needs of the target population. Similarly, studies from Norway and France highlighted the importance of incorporating local cultural nuances into the instruments. These adaptations included careful consideration of local healthcare practices, patient-provider communication styles, and specific cultural attitudes toward health and illness [26]. Such detailed reporting on cultural adaptations underscores the importance of ensuring that PREMs are linguistically accurate and culturally relevant.

Another key objective of the review was to examine the psychometric properties of the selected instruments and reveal important insights into the robustness and reliability of the tools used to assess patient-reported experiences and outcomes in diabetes mellitus (DM) care. Six of the eight instruments were tested for reliability, and validity which are critical properties for ensuring the instruments provide consistent and accurate measurements [24–29]. Most instruments demonstrated acceptable internal consistency, with Cronbach’s α values exceeding 0.70. However, the absence of internal consistency testing in the remaining instruments raises concerns about their reliability and the consistency of the data they produce [40]. Furthermore, while Cronbach’s alpha values were reported for the overall instrument, they were not provided for each specific dimension. This lack of dimension-level reliability data means that the consistency of responses within individual dimensions is uncertain, potentially impacting the accuracy and interpretability of results when assessing specific aspects of patient experience [41]. The instrument developed in Ecuador stands out as the most rigorously tested, having undergone evaluations for internal consistency, test-retest reliability, face validity, content validity, construct validity, and responsiveness [29]. In contrast, some instruments lack psychometric testing, making it difficult to assess their reliability, validity, and responsiveness, which weakens confidence in the accuracy and consistency of the data they produce [40].

This scoping review has some limitations that should be acknowledged. Firstly, the review was limited to articles published in English, potentially leading to language bias, and relevant studies published in other languages were excluded. While major popularly used databases such as PubMed, Embase, CINAHL, Cochrane, and Scopus were searched, there is a possibility that other relevant databases and grey literature sources were not included, which might have led to the omission of pertinent studies. Lastly, the review included a relatively small number of studies (eight articles), which reflects the limited availability of research on this topic. This small sample size may affect the robustness and generalizability of the conclusions drawn. Another limitation is the lack of dimension-level reliability testing for some of the included instruments, which raises concerns about the internal consistency of specific dimensions. This gap limits the ability to assess the interpretability and reliability of the tools for measuring specific aspects of patient experience in diabetes care. Addressing these limitations provides a transparent and balanced perspective on the strengths and weaknesses of this scoping review, enhancing its credibility and utility for future research.

## Conclusion and implications

This scoping review summarizes the different tools specifically designed to assess PREMs for patients living with diabetes care across various countries. Future research should address several key areas to enhance the development of tools to measure PREMs to enhance diabetes care. First, exploring patient experiences in more diverse settings, whether in geographical settings or among inpatient settings, is encouraged. Culturally relevant measures should be investigated to reflect diverse healthcare contexts and enhance the accuracy of patient-reported outcomes. With different domains being measured, the review identified five overarching domains, representing a step toward standardizing the assessment of diabetes care from the patient’s perspective. Furthermore, evaluating the feasibility and acceptability of both new and existing tools can help ensure their practical application and effectiveness. Lastly, longitudinal studies are also needed to assess the stability and responsiveness of these instruments over time.

## Electronic supplementary material

Below is the link to the electronic supplementary material.

Supplementary Material 1

Supplementary Material 2

Supplementary Material 3

## Data Availability

All data generated or analysed during this study are included in this published article [and its supplementary information file.
