# Supplementary material for: Instruments for assessing patient-reported experience measures among patients with diabetes mellitus: a scoping review
Source: J Patient Rep Outcomes. 2025 Feb 8;9:16. doi: 10.1186/s41687-025-00848-7 (PMC11807032; doi:10.1186/s41687-025-00848-7)
Supplement: Supplementary file 2 — Supplementary Material 2 [file 41687_2025_848_MOESM2_ESM.pdf]

Final Literature Search Strategies (Additional File 1)

Literature search methods:

The following search strategy was applied across five databases: PubMed, Scopus, Cochrane, CINAHL, and Embase. The search was conducted to identify studies related to diabetes mellitus (type 1 and type 2) and patient-reported experience measures (PREMs). No restrictions were placed on the year of publication, but the search was limited to English-language publications.

Search Strategy Across Databases

Databases Searched:

- PubMed
- Scopus
- Cochrane
- CINAHL
- Embase

Filters Applied:

Language: English

Year of Publication: Not Limited

Search Terms and Steps:

The following steps were used in each database with slight adjustments for their indexing terms and syntax (e.g., MeSH for PubMed). In Embase, a regular keyword search was used, and relevant studies were identified based on the appearance of keywords related to diabetes and patient-reported experience measures.

PubMed Search Strategy

Steps	Words	Results
#1	"Diabetes Mellitus, Type 2"[Mesh]	176,267
#2	"Diabetes Mellitus, Type 1"[Mesh]	87,662
#3	#1 OR #2	247,087
#4	"Patient-reported experience"	644
#5	"Patient-reported experience measures"	343
#6	"PREM"	1,252
#7	#4 OR #5 OR #6	1,745
#8	#3 AND #7<br>(DM AND PREM)	26

Scopus Search Strategy

Steps	Words	Results
#1	"Diabetes Mellitus, Type 2"	163,785
#2	"Diabetes Mellitus, Type 1"	63,158
#3	#1 OR #2	213,804
#4	"Patient-reported experience"	3,326
#5	"Patient-reported experience measures"	1,380
#6	"PREM"	45,804
#7	#4 OR #5 OR #6	48,783
#8	#3 AND #7<br>(DM AND PREM)	136

Embase Search Strategy

Steps	Words	Results
#1	"Diabetes Mellitus, Type 2"	361,043
#2	"Diabetes Mellitus, Type 1"	152,073
#3	#1 OR #2	477,423
#4	"Patient-reported experience"	1,022
#5	"Patient-reported experience measures"	465
#6	"PREM"	8,514
#7	#4 OR #5 OR #6	9,272
#8	#3 AND #7<br>(DM AND PREM)	144

Cochrane Search Strategy

Steps	Words	Results
#1	"Diabetes Mellitus, Type 2"[Mesh]	23,599
#2	"Diabetes Mellitus, Type 1"[Mesh]	6,900
#3	#1 OR #2	29,509
#4	“Patient-reported experience”	120
#5	“Patient-reported experience measures”	46
#6	“PREM”	93
#7	#4 OR #5 OR #6	182
#8	#3 AND #7<br>(DM AND PREM)	2

CINHAL Search Strategy

Steps	Words	Results
#1	"Diabetes Mellitus, Type 2"[Mesh]	13,806
#2	"Diabetes Mellitus, Type 1"[Mesh]	24,850
#3	#1 OR #2	30,030
#4	“Patient-reported experience”	37
#5	“Patient-reported experience measures”	19
#6	“PREM”	1518
#7	#4 OR #5 OR #6	1,550
#8	#3 AND #7<br>(DM AND PREM)	47

Explanation of Search Terms:

#1 Diabetes Mellitus, Type 2: This search term was used to identify studies related to type 2 diabetes mellitus across all databases. In PubMed, the MeSH term was used

#2 Diabetes Mellitus, Type 1: This search term was used to identify studies related to type 1 diabetes mellitus.

#3 Combined search term for both type 1 and type 2 diabetes mellitus

#4 Patient-reported experience: This term was used to capture studies focusing on patient-reported experience in healthcare.

#5, #6 Patient-reported experience measures (PREMs): This term was used to further specify studies involving the use of validated patient-reported experience measures in healthcare settings.

#7 Combined Search for Patient-Reported Experience Terms

#8 Combined Search of Diabetes Mellitus and Patient-Reported Experience Measures

Results Overview:

The search strategy yielded a total of 136 relevant studies in PubMed. The number of studies retrieved from each of the other databases is listed under each respective search strategy
