# Supplementary material for: Instruments for assessing patient-reported experience measures among patients with diabetes mellitus: a scoping review
Source: J Patient Rep Outcomes. 2025 Feb 8;9:16. doi: 10.1186/s41687-025-00848-7 (PMC11807032; doi:10.1186/s41687-025-00848-7)
Supplement: Supplementary file 3 — Supplementary Material 3 [file 41687_2025_848_MOESM3_ESM.docx]

| **Additional File 2: Detailed Descriptions of Original Domains and Summary of Commonly Measured Domains in PREMs** | | | | | | | | |
| --- | --- | --- | --- | --- | --- | --- | --- | --- |
| **No** | **Instrument** | **Domain** | **Subscales(Items)** | **Care planning** | **Patient education** | **Professionalism** | **Quality of service** | **Hospital care & transition** |
| **1** | **PEDS [14]** | **Care planning (9)** | How many times have you visited the care provider for diabetes care? | √ |  |  |  |  |
|  |  |  | Did you discuss your ideas and goals about the best way to manage your diabetes with the clinic staff? | √ |  |  |  |  |
|  |  |  | Did you discuss the food you eat and any changes you could make to your diet? | √ |  |  |  |  |
|  |  |  | Did you discuss your levels of physical activity and any changes that you could make? | √ |  |  |  |  |
|  |  |  | During your last diabetes appointment, did you discuss and agree on a plan about how to manage your diabetes until your next appointment? | √ |  |  |  |  |
|  |  |  | Were you offered a written, printed, or electronic copy of your care plan? | √ |  |  |  |  |
|  |  |  | Do you feel the health professional listened carefully to what you had to say? |  |  | √ |  |  |
|  |  |  | Did the health professional explain things clearly? |  |  | √ |  |  |
|  |  |  | As a result of your diabetes appointment in the last 12 months, do you feel confident about managing your diabetes? | √ |  |  |  |  |
|  |  | **Care provision (3)** | Sometimes, one health professional will say one thing and another will say something quite different. In the last 12months, has this happened to you? |  |  |  | √ |  |
|  |  |  | In the last 12 months, have you needed to provide the same important information to clinic staff that you had already provided during a previous visit? |  |  |  | √ |  |
|  |  |  | Do you have a contact number to call if you are worried about your diabetes? |  |  |  | √ |  |
| **2** | **Patient-reported experience measure for young people with type 1 diabetes [15]** | **Learning new information (4)** | Learning through processing and understanding clinical results |  | √ |  |  |  |
|  |  |  | Observing change and the effects |  | √ |  |  |  |
|  |  |  | Learning new things from multiple members of staff and leaflets |  | √ |  |  |  |
|  |  |  | Learning new things through meeting other young people |  | √ |  |  |  |
|  |  | **Age appropriateness (2)** | Clinic environment, waiting times and activities |  |  |  | √ |  |
|  |  |  | Communication |  |  |  | √ |  |
| **3** | **NDR-Swedish [17]** | **Support from diabetes care provider (9)** | Do you get the support you need from your diabetes care provider? |  |  |  | √ |  |
|  |  |  | Is it easy to contact your diabetes care provider when you need help with your diabetes? |  |  |  | √ |  |
|  |  |  | Are you able to see a nurse as often as you feel is necessary for your diabetes? |  |  |  | √ |  |
|  |  |  | Are you able to make visits with your nurse that fit your schedule? |  |  |  | √ |  |
|  |  |  | Are you able to see the same nurse for your diabetes at every visit? |  |  |  | √ |  |
|  |  |  | Are you able to see a doctor as often as you feel is necessary for your diabetes? |  |  |  | √ |  |
|  |  |  | Are you able to make visits with your doctor that fit your schedule? |  |  |  | √ |  |
|  |  |  | Are you able to see the same doctor for your diabetes at every visit? |  |  |  | √ |  |
|  |  |  | Are you able to talk about matters that are important to you at the appointments about your diabetes? |  | √ |  |  |  |
|  |  | **Medical devices and medical treatment (3)** | How satisfied are you with the medical devices available for you to monitor your blood sugar level? | √ |  |  |  |  |
|  |  |  | How satisfied are you with the medical devices that you have available for you to take insulin (for example, an insulin pen or insulin pump0? | √ |  |  |  |  |
|  |  |  | How satisfied are you with your medication treatment? The question includes all your medications you take. | √ |  |  |  |  |
| **4** | **APEQ-DC [19]** | **Consultation (7)** | Do you get good training in how to use the equipment? |  | √ |  |  |  |
|  |  |  | Are you greeted well when you arrive at the pediatric department? |  |  |  | √ |  |
|  |  |  | Do you get good help choosing the right insulin dosage? |  | √ |  |  |  |
|  |  |  | Are you involved in deciding what to follow up before the next consultation? | √ |  |  |  |  |
|  |  |  | Does it seem that those working at the outpatient clinic understand what it’s like to be young and have diabetes? |  |  |  | √ |  |
|  |  |  | Do you have enough time in the consultation with the doctor or nurse? |  | √ |  |  |  |
|  |  |  | Are you involved in deciding what equipment to use? | √ |  |  |  |  |
|  |  | **Information on food and physical activity/exercise (2)** | Do you get good information and counselling on food intake? | √ | √ |  |  |  |
|  |  |  | Do you get good information and counselling on physical activity/exercise? | √ | √ |  |  |  |
|  |  | **Nurse contact (3)** | Do the nurses talk to you in a way you understand? |  |  | √ |  |  |
|  |  |  | Does it seem like the nurses are knowledgeable about diabetes and diabetes care? |  |  | √ |  |  |
|  |  |  | Do you feel safe raising difficult issues with the nurses? |  |  | √ |  |  |
|  |  | **Doctor contact (3)** | Does the doctor talk to you in a way you understand? |  |  | √ |  |  |
|  |  |  | Do you feel safe raising difficult issues with the doctor? |  |  | √ |  |  |
|  |  |  | Does it seem like the doctor is knowledgeable about diabetes and diabetes care? |  |  | √ |  |  |
|  |  | **Outcome (1)** | Overall, has the follow-up at the outpatient clinic helped you with your diabetes |  |  |  | √ |  |
| **5** | **Patient-reported experience measures for patient satisfaction with outpatient clinic [20]** | **Satisfaction with the outpatient clinic (1)** | Satisfaction with the outpatient clinic |  |  |  | √ |  |
|  |  | **Benefit (3)** | Benefit consultation |  |  |  | √ |  |
|  |  |  | Unnecessary visits |  |  |  | √ |  |
|  |  |  | Needs covered |  |  |  | √ |  |
|  |  | **Accessibility (3)** | Contact when needed |  |  |  | √ |  |
|  |  |  | Appointment when needed |  |  |  | √ |  |
|  |  |  | Means of contact |  |  |  | √ |  |
| **6** | **EDP questionnaire [21]** | **Information**  **(3)** | I have received information about the exercise I can do | √ |  |  |  |  |
|  |  |  | I have received information in words that I could understand |  | √ |  |  |  |
|  |  |  | I have learned to cope with my diabetes |  | √ |  |  |  |
|  |  | **Care delivery (2)** | I have been able to talk to the doctor about what is important to me |  |  | √ |  |  |
|  |  |  | I can contact my doctor whenever I need to |  |  |  | √ |  |
|  |  | **Patient-centered care (3)** | The doctor has explained to me what can I eat | √ |  |  |  |  |
|  |  |  | I felt that the doctor listened to me in the consultation |  |  | √ |  |  |
|  |  |  | I am prepared to know what to do in case something unexpected happens with my diabetes | √ |  |  |  |  |
| **7** | **Patient-reported experience measure for adult inpatient diabetes care [16]** | **Admission to hospital(2)** | When you first went into the hospital, did the healthcare staff know about your diabetes? |  |  |  |  | √ |
|  |  |  | Did you get the chance to tell the healthcare staff what you do to keep your glucose in your target range? | √ |  |  |  |  |
|  |  | **Managing your diabetes during your hospital stay (4)** | Did you continue checking your blood glucose levels when you were in the hospital? |  |  |  |  | √ |
|  |  |  | Did you continue managing your diabetes with diet when you were in the hospital? |  |  |  |  | √ |
|  |  |  | Did you continue managing your diabetes by being active and moving enough when you were in the hospital? |  |  |  |  | √ |
|  |  |  | Did you receive foot care when you were in the hospital? |  |  |  |  | √ |
|  |  | **Medication and equipment (6)** | Was there a change to your diabetes medication during your hospital stay? |  |  |  |  | √ |
|  |  |  | Were you given enough information by healthcare staff about how to use your new diabetes medication? |  | √ |  |  |  |
|  |  |  | Did the healthcare staff discuss with you why there was a change to your diabetes medication? |  | √ |  |  |  |
|  |  |  | Were you given any diabetes equipment for you to use which was new to you when you were in the hospital? | √ |  |  |  |  |
|  |  |  | Were you told or shown by healthcare staff how to use this new diabetes equipment? |  | √ |  |  |  |
|  |  |  | Were you confident in using this new diabetes equipment when you got home? |  | √ |  |  |  |
|  |  | **Treatment and care (8)** | If your blood glucose levels were too high or too low in the hospital, was this dealt with quickly enough by healthcare staff? |  |  |  |  | √ |
|  |  |  | Was the reason for your blood glucose levels being too high or too low discussed with you? | √ |  |  |  |  |
|  |  |  | Did you have confidence in the decisions made about your diabetes during your hospital stay? | √ |  |  |  |  |
|  |  |  | Were you involved in decisions about your diabetes during your hospital stay? |  |  |  |  | √ |
|  |  |  | Did you have enough information and support from healthcare staff to make decisions about your diabetes care in hospital? |  |  |  |  | √ |
|  |  |  | Were you confident that the healthcare staff looking after you in the hospital were: -a) Checking your blood glucose levels at the right times? | √ |  |  |  |  |
|  |  |  | Were you confident that the healthcare staff looking after you in the hospital were: -b)Giving you your diabetes medication (including tablets or insulin) at the right times? |  |  | √ |  | √ |
|  |  |  | Were you confident that the healthcare staff looking after you in the hospital were: -c) Changing how your diabetes was managed if your blood glucose levels became too high or too low? | √ |  |  |  |  |
|  |  |  | Did you have confidence and trust in the following healthcare staff managing your diabetes during your hospital stay? -a)Ward nursing staff? |  |  | √ |  | √ |
|  |  |  | Did you have confidence and trust in the following healthcare staff managing your diabetes during your hospital stay? b)Diabetes nurse specialists? |  |  | √ |  | √ |
|  |  |  | Did you have confidence and trust in the following healthcare staff managing your diabetes during your hospital stay?c)Ward doctors? |  |  | √ |  | √ |
|  |  |  | Did you have confidence and trust in the following healthcare staff managing your diabetes during your hospital stay?d)Diabetes specialist doctors? |  |  | √ |  | √ |
|  |  |  | Were healthcare staff sensitive to your emotional needs and feelings relating to your diabetes during your hospital stay? |  |  |  |  | √ |
|  |  | **Communication (5)** | Was there enough communication between the healthcare staff and you about your blood glucose levels and diabetes treatment when in the hospital? |  |  |  |  | √ |
|  |  |  | When healthcare staff gave you information about your diabetes when in hospital, did you understand this? |  |  |  |  | √ |
|  |  |  | If you had any questions about your diabetes during your hospital stay, did you get answers that you understood? |  |  |  |  | √ |
|  |  |  | Sometimes in the hospital, a member of staff will say one thing and another will say something quite different. Did this happen to you about your diabetes? |  |  |  |  | √ |
|  |  |  | Did you ever have to repeat information about your diabetes to different healthcare staff? |  |  |  | √ |  |
|  |  | **Hospital Food (5)** | Did you have any hospital food during your stay? |  |  |  |  | √ |
|  |  |  | During your stay in the hospital, were you offered food choices that were suitable to your diabetes? |  |  |  |  | √ |
|  |  |  | During your stay in the hospital, did you have access to meals and food at times that were suitable for managing your diabetes (outside of meal times)? |  |  |  |  | √ |
|  |  |  | Was the food appetizing? |  |  |  |  | √ |
|  |  |  | Were you given enough information about the hospital meals to make appropriate choices about what to eat? |  |  |  |  | √ |
|  |  | **Leaving Hospital (1)** | Before you left the hospital, were you given information about how to manage your diabetes when you got back home? |  |  |  |  | √ |
| **8** | **DREAMS [18]** | **Relationship with the physician (4)** | I feel listened to |  |  | √ |  |  |
|  |  |  | My choices (treatments, lifestyle, etc.) are respected |  |  | √ |  |  |
|  |  |  | I am treated with respect and dignity |  |  | √ |  |  |
|  |  |  | My physician makes sure that I clearly understand the medical information they provide during the consultation (definitions of disease and complications, treatments’ mode of action, dose etc.) |  |  | √ |  |  |
|  |  | **Medical care experience (4)** | In between consultations, my physician makes sure I understand what I must do to manage my diabetes  (consultation with other specialists, check-ups, taking treatments, monitoring, etc.) |  |  | √ |  |  |
|  |  |  | My physician brings up nutrition as a part of my diabetes management | √ |  |  |  |  |
|  |  |  | My physician brings up physical activity as a part of my diabetes management | √ |  |  |  |  |
|  |  |  | My physician brings up the psychological impact of diabetes on my daily life (mental load, anxiety, etc.) | √ |  |  |  |  |
|  |  | **Illness appropriation (3)** | I know all my treatments and why they were prescribed |  | √ |  |  |  |
|  |  |  | I know the potential side effects of my treatments |  | √ |  |  |  |
|  |  |  | I know the possible complications of diabetes |  | √ |  |  |  |
|  |  | **Medical consultation (3)** | It is easy for me to book an appointment (reach the physician, inadequate notice, waiting time, etc.) |  |  |  | √ |  |
|  |  |  | The consultation takes place on time |  |  |  | √ |  |
|  |  |  | There are no interruptions during the consultation (phone calls, outside requests, etc.) |  |  |  | √ |  |
|  |  | **Care structure (4)** | When you arrive, the reception at the care structure is |  |  |  | √ |  |
|  |  |  | The medical team is ?? |  |  |  | √ |  |
|  |  |  | The accessibility of the care structure (transportation, parking, standards for people with reduced |  |  |  | √ |  |
|  |  |  | mobility) is ?? |  |  |  | √ |  |
|  |  |  | I would recommend the care structure to another person with a diabetes similar to mine: |  |  |  | √ |  |
